# Trans-splenic percutaneous glue embolization of bleeding gastric varices in the setting of malignant sinistral portal hypertension

**DOI:** 10.1186/s42155-024-00467-1

**Published:** 2024-07-27

**Authors:** Ken Zhao, Sam Y. Son, Debkumar Sarkar, Ernesto G. Santos

**Affiliations:** Department of Radiology, Memorial Sloan Kettering Cancer Center, New York, NY USA

**Keywords:** Trans-splenic percutaneous glue embolization, Gastric varices, Sinistral portal hypertension

## Abstract

Sinistral portal hypertension, also known as left-sided portal hypertension, is a rare cause of gastric variceal bleeding which occurs secondary to occlusion of the splenic vein. We present a case of venous occlusion and sinistral portal hypertension secondary to distal pancreatic cancer requiring treatment of gastric variceal bleeding. After failing conservative management, transvenous intervention was attempted, but a venous communication with the gastric varices was unable to be identified on multiple venograms. A percutaneous trans-splenic approach using a 21-G needle and ultrasound guidance was successful in directly accessing an intraparenchymal vein feeding the gastric varices, and glue embolization was performed directly through the access needle with excellent results.

## Background

Sinistral portal hypertension (SPH), also known as left-sided portal hypertension, is a rare cause of gastric variceal bleeding which occurs secondary to occlusion of the splenic vein. The compromised venous outflow results in splenic venous hypertension and formation of decompressive collaterals that can become gastric varices. The treatment approach for variceal bleeding due to SPH differs markedly from variceal bleeding due to conventional right-sided portal hypertension, where the mainstay of radiologic treatment involves transjugular intrahepatic portosystemic shunt (TIPS) creation or balloon-occluded retrograde transvenous obliteration (BRTO) [1, 2]. Instead, splenic venous recanalization, partial splenic embolization, splenic arterial embolization, or surgical splenectomy are recommended. Endoscopic management with ligation or obturation by glue injection can be effective. However, gastric varices can be challenging to endoscopically ligate and glue obturation requires operator expertise, as delivery of liquid embolic may be complicated by systemic embolization [3]. Transvenous variceal sclerosis, such as BRTO, is an option but requires a catheterizable venous communication and may precipitate splenic congestion [4]. Creation of a TIPS would be ineffective unless there is concomitant right-sided portal hypertension contributing to the gastric varices. We report a case of gastric variceal bleeding in the setting of malignant SPH treated with direct trans-splenic percutaneous glue embolization.

## Case report

A patient with metastatic distal pancreatic cancer invading into the splenic hilum, resulting in malignant splenic venous occlusion and SPH with gastric varices, presented with anemia and recurrent melena. The patient’s first presentation with gastric variceal bleeding was 17 months prior, at which time coil embolization of the mid-splenic artery was performed. The tumor’s hilar location prohibited venous recanalization and stent placement.

**Fig. 1:**
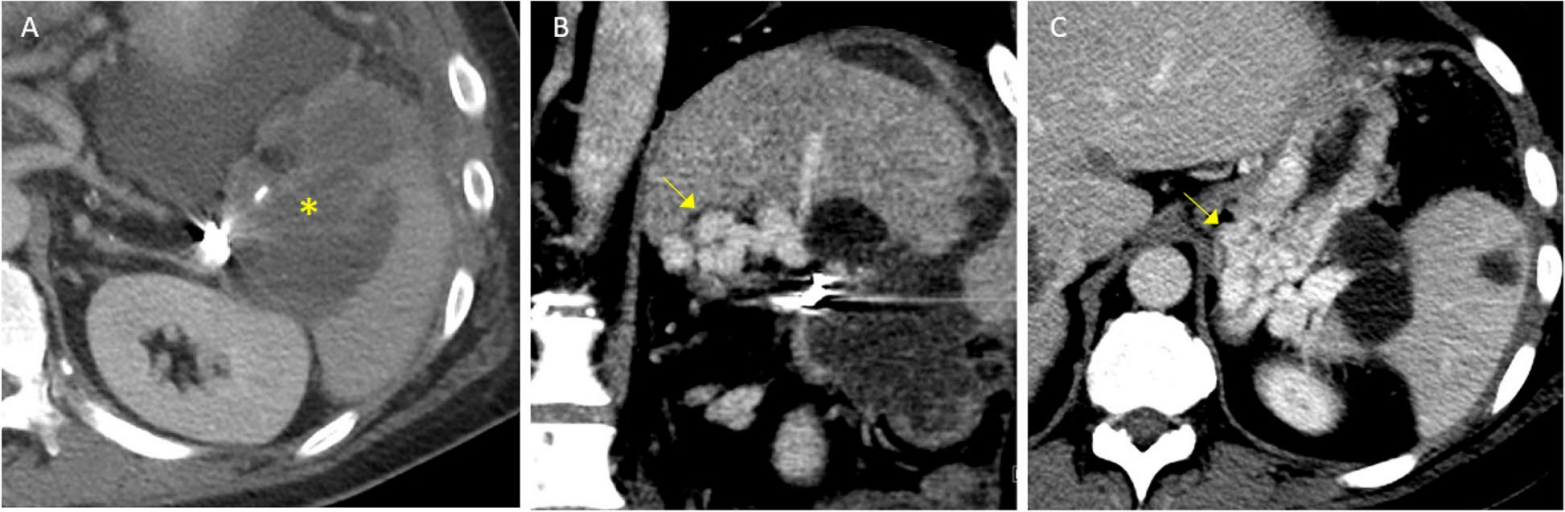
Pre-procedural contrast-enhanced CT. **A** Axial image shows the pancreatic tail neoplasm invading the splenic hilum (asterisk) and adjacent coils from prior splenic artery embolization. **B** Coronal image shows large gastric fundal varices (arrow). Note location of the varices relative to the coil pack. **C** Axial image shows large gastric fundal varices (arrow)

Contrast-enhanced CT visualized large gastric fundal varices (Fig. 1). These varices were confirmed by endoscopy and exhibited red wale signs suggesting recent hemorrhage. Conservative management was attempted, but the patient continued to have melena and transfusion dependent anemia. Gastroenterology deferred endoscopic intervention, and the patient was deemed a poor surgical candidate due to comorbidities.

The case was reviewed during interventional radiology rounds. Creation of a TIPS was excluded because there was neither cirrhosis nor stigmata of right-sided portal hypertension. Further splenic arterial embolization was excluded because the prior coil embolization precludes selection and embolization of sufficient vascular territory.

**Fig. 2:**
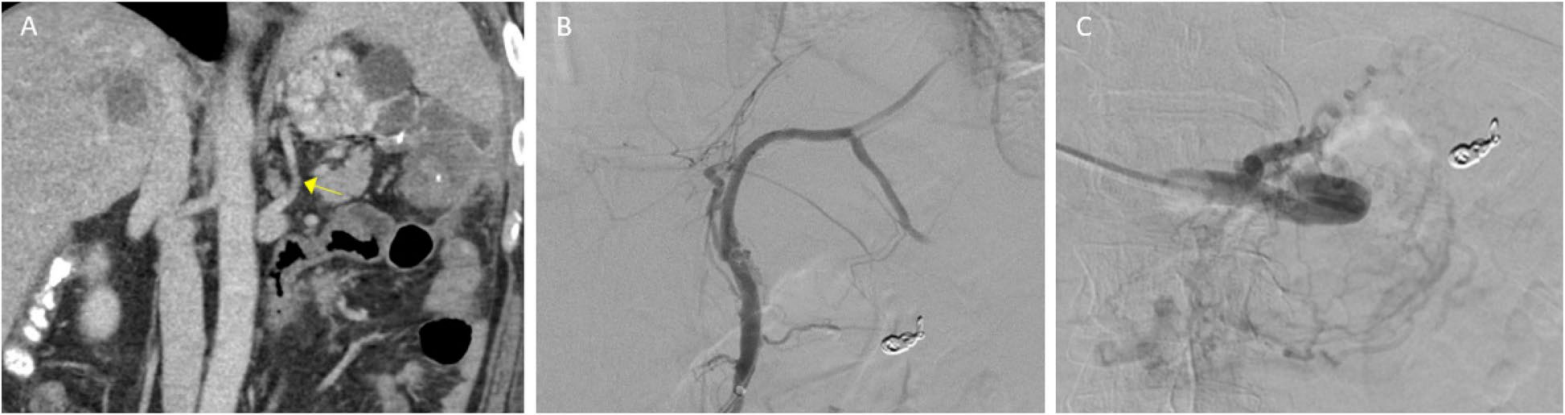
**A** Pre-procedural contrast-enhanced CT shows suspected gastro-renal communication with the gastric varices (arrow). **B** Venogram of the suspected shunt shows no communication with the gastric varices, only prominent diaphragmatic veins. **C** Left gastric balloon occlusion venogram shows no communication with the gastric varices. Normal appearing gastric parenchymal veins

A suspected gastro-renal shunt ascending from the left renal vein visualized on the pre-procedural CT, and BRTO was first attempted. This suspected shunt was selected with a 6 Fr sheath, but venography did not demonstrate communication with the gastric varices, only prominent diaphragmatic drainage. Balloon occlusion venography was not performed because the sheath nearly occluded the ascending vein, and the contrast column was stagnant without washout (Fig. 2). Balloon placement would have been the next step if further interrogation was desired.

Percutaneous transhepatic portal venous access was subsequently obtained. A prominent left gastric vein, seen on the recent CT, was selected with a 5.5 Fr balloon occlusion catheter. Balloon occlusion venography visualized no gastric varices, only normal gastric parenchymal veins (Fig. 2).

**Fig. 3:**
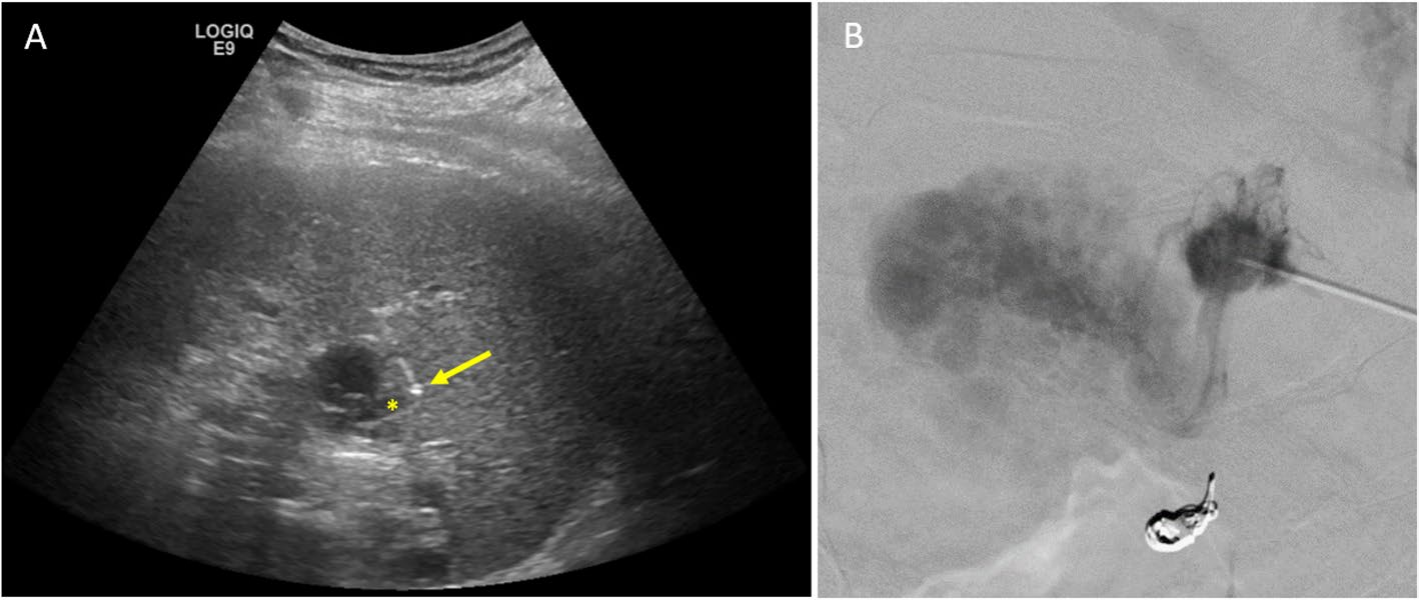
Gastric variceal venogram via direct puncture. **A** Puncture of an intraparenchymal splenic vein (asterisk) using a 21-gauge access needle (arrow). **B** Hand injection venogram through the access needle opacifies the gastric varices. No prominent draining vein seen

**Fig. 4:**
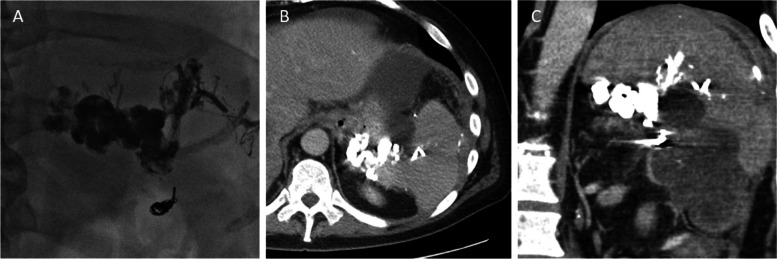
Completion images. **A** The gastric varices are well filled with glue cast. **B** Axial and **C**. coronal views from a post-procedural CT visualizing the embolized gastric varices

Ultrasound guided trans-splenic puncture with a 21-G needle was then used to directly access an intraparenchymal vein supplying the gastric varices (Fig. 3A). Hand-injection venography through the 21-G needle visualized the gastric varices without any large draining veins (Fig. 3B). There was mild parenchymal staining with contrast at the needle tip, but flow of contrast into the vein and varices was brisk. Based on the contrast volume, injection duration required to opacify the varices, and flow-rate within the varices, 5 mL of 1:4 ratio glue (Trufill, Johnson & Johnson, New Brunswick, New Jersey) and lipiodol was used to embolize the varices via injection through the access needle. The glue cast filled the varices, and no distal embolization occurred (Fig. 4). The splenic tract was embolized as the needle was withdrawn using the trace residual glue within the needle and syringe.

Post-procedurally, the patient’s melena resolved, and hemoglobin remained stable. There were no complications. The patient was discharged on post-procedural day 4. There were no further episodes of melena during the remainder of the patient’s life, which was 3 months due to metastatic pancreatic cancer.

## Conclusion

Percutaneous trans-splenic glue embolization is similar to endoscopic variceal obturation by glue injection. However, interventional radiologists routinely utilize fluoroscopy and perform venography. This facilitates accurate characterization of variceal anatomy, variceal flow dynamics, and identification of potential draining veins, decreasing the potential for systemic non-target embolization. Percutaneous trans-splenic glue embolization should be considered for cases of bleeding gastric varices due to sinistral portal hypertension.

## Data Availability

Data sharing is not applicable to this article as no datasets were generated or analyzed during the current study.

## Abbreviations

SPH: Sinistral portal hypertension
TIPS: Transjugular intrahepatic portosystemic shunt
BRTO: Balloon-occluded retrograde transvenous obliteration
